# Impact of COVID-19 on Early Childhood Educator’s Perspectives and Practices in Nutrition and Physical Activity: A Qualitative Study

**DOI:** 10.1007/s10643-021-01195-0

**Published:** 2021-04-24

**Authors:** Lynne Lafave, Alexis D. Webster, Ceilidh McConnell

**Affiliations:** Mount Royal University, Calgary, AB Canada

**Keywords:** Preschool, Early childhood education and care, COVID-19, Healthy eating, Physical activity, Qualitative description

## Abstract

Government guidelines for relaunching early childhood education and care (ECEC) programs during the COVID-19 pandemic have required the implementation of various practices to minimize the risk of infection transmission. These directives include recommendations regarding serving and handling food, shared spaces, and physical distancing which have a direct impact on the health and development of children in care. The purpose of this study was to explore early childhood educators’ perspectives on how COVID-19 guidelines have impacted the nutrition and physical activity practices within their ECEC environment. A qualitative description approach was used to explore a purposive sample of 17 educators working full time in ECEC centres during the pandemic between July and August 2020. Semi-structured, individual interviews were conducted, audio-recorded, transcribed verbatim, and analysed using a thematic analysis. Educators identified environmental changes in their environments noting a reduction in the quality food available for children juxtaposed with increased outdoor opportunities and a perceived increase in physical activity time. Teaching practices were also identified as being impacted by the COVID-19 guidelines. Curriculum, life skills, and social connectedness around healthy eating education was disrupted. Promoting physical activity education was challenged due to equipment, space, and curriculum enrichment opportunities losses. These findings demonstrate pronounced negative effects of COVID guidelines on nutrition and physical activity best practices within the ECEC environment. There is a need to support educators in maintaining healthy and active environments for preschoolers while following guidelines to minimize the risk of COVID infection transmission.

## Introduction

The purpose of the present article is to explore early childhood educators’ perspectives on how COVID-19 guidelines have impacted healthy eating and physical activity in child care environments. The rationale of the study is based on (1) the role of child care experiences in shaping healthy eating and physical activity behaviours for lifelong well-being, and (2) the impact of COVID-19 guidelines on the early childhood education and care environment practices.

Early childhood, birth to five years, is a period of significant cognitive, social, and physical growth and development [Goldfield et al., 2012; World Health Organization (WHO), 2019]. The formation of healthy eating habits and regular physical activity established during early childhood tend to persist into adulthood (WHO, 2016). As a result, education and care environments in the early years have powerful impacts on the healthy behaviour choices of future adults, which then can impact the prevention of chronic diseases (Herman et al., 2009; WHO, 2016). In Canada, approximately 60% of children under the age of five receive child care outside the family home (Statistics Canada, 2020). In addition, families who enroll their children in child care tend to do so on a full-time basis; that is, 70% of children enrolled in early childhood education and care (ECEC) programs attend for a minimum of 30 h per week (Sinha, 2014). ECEC centers provide young children with social, eating, and physical activity opportunities that support healthy development (Gable & Lutz, 2001; Hinkley et al., 2012).

### The Influence of the ECEC Environment on a Child’s Relationship with Food

The ECEC nutrition environment is an interplay between the foods available for children throughout the day along with the education connections surrounding that food to support healthy development. Early childhood is a critical time for food literacy education as children explore food preferences and are particularly responsive to healthy messaging (Parletta, 2014). Mealtimes, the eating environment, and nutrition activities are all key opportunities within the ECEC context to guide and teach children about healthy food choices in addition to supporting the development of positive food attitudes. Mealtime is an opportunity to model health promoting behaviours and to engage children in rich conversations which promote social development (Dev et al., 2014; Swinburn et al., 2013). Family-style meal service (FSMS) in child care can promote a healthy eating environment. FSMS involves children serving themselves from communal dishes placed on the table, this allows children to choose what and how much to eat from the dishes offered, thus helping children understand their hunger cues [Alberta Health Services (AHS), 2018]. FSMS plays an important role in supporting children’s self-regulation, motor development by learning how to serve themselves, and the development of social skills (Benjamin Neelon & Briley, 2011; Dev et al., 2014; Fletcher, Branen, & Price, 2005). Moreover, incorporating nutrition education and skills into child care curricula reinforces children’s positive attitudes toward healthy eating thus supporting lifelong healthy eating habits (Government of Alberta, 2012; Utter et al., 2018).

### The Influence of the ECEC Environment on a Child’s Activity

The World Health Organization (WHO) has developed physical activity guidelines for preschool aged children (3–5 years) that recommends an overall 180 min of daily physical activity and 60 min of that should be devoted to energetic play (Willumsen & Bull, 2020). Activities in child care that can support the development of gross and fine motor skills may include indoor and outdoor play, access to playing with a variety of equipment, and community programming such as field trips or community expert-led classes. An important contributor to increasing young children’s physical activity while attending child care is having access to both indoor and outdoor spaces that allow for active play (Cardon, Van Cauwenberghe, Labarque, Haerens, & De Bourdeaudhuij, 2008; Gubbels et al., 2012). Additionally, children who have access to more indoor play equipment and frequently go on field trips tend to be more physically active (Dowda et al., 2004; Gubbels et al., 2012).

### COVID-19 and the ECEC Environment

On March 11, 2020 the WHO declared the COVID-19 outbreak as a global pandemic (WHO, 2020). Governments across the world implemented various physical distancing restrictions to prevent the spread of COVID-19, resulting in economic shutdowns and disruptions to everyday life. Initially, the Alberta government called for the widespread shutdown of ECEC centers; however, by early April select programs were allowed to reopen to provide child care for essential workers. In mid-May, all ECEC centers were permitted to resume under specific guidelines developed to prevent the spread of the virus (Government of Alberta, 2020). The guidelines include restrictions on physical distancing and visitation, establishment of cohorts, sanitization protocols, and food-handling procedures. Additional public health orders limited access to shared public spaces and discontinued community programming. Health promotion activities such as gardening, playgrounds, and learning centers became inaccessible. COVID-19 guidelines directly impact ECEC environments and, consequently, the healthy development of children in care.

### Research Questions

The purpose of the present study is to explore educators’ perspectives on how COVID-19 guidelines have impacted healthy eating and physical activity in child care environments. A comprehensive understanding of the change in center practices that educators are facing in response to the pandemic is necessary to inform future public health interventions.

The following research questions were addressed:

- (1) How have COVID-19 guidelines impacted the nutrition and physical activity environment in the early childhood education?
- (2) What changes do educators report making to their teaching practices related to nutrition and physical activity in response to the COVID-19 guidelines?

## Methods

### The Critical Ecology Framework

Given the complexity of factors and systems governing decisions and actions within the ECEC context, we adopted a critical ecology of the early childhood profession framework in our research approach (Dalli, Miller, & Urban, 2012). This framework, building upon Bronfenbrenner’s (1979) ecological systems theory, addresses unequal power relationships by placing practitioners at the center of the model to ensure that their voices are essential in the research conversation. As a central tenet, people’s behaviours are influenced by relationships and social interactions. The use of this framework is particularly appropriate for studies aiming to address the voices of ECEC practitioners and places emphasis on ECE practitioners as producers of knowledge (Baker, 2018).

Dalli and colleagues (2012) places the practitioner and those interacting daily in microsystems, at the center of the framework. The mesosystem explains relationships and connections between multiple microsystems. The next outer layer is the exosystem, where educators do not participate but decisions are made that affect them directly. This is followed by the macrosystem which encompasses cultural values and socio-political context. Lastly, the chronosystem contextualizes developments in the system over time. These systems nest within each other with the microsystems at the core.

### Research Design

A qualitative approach for this study was chosen to respect and acknowledge the emic or insider perspective of early childhood educators as front-line workers in the COVID-19 pandemic. Qualitative description design provides an avenue to understand a phenomenon through the meanings attributed to them by the participants (Bradshaw et al., 2017). This design aligns well with the purpose of this study by providing insight into the impact of the COVID pandemic on the eating and physical activity environment within ECEC programs. The semi-structured, individual interviews facilitate a conversational approach, eliciting richer descriptions regarding the participants’ understanding and attitudes in a manner that provides the space for participants to express themselves freely (Kallio et al., 2016). All procedures in this research study were submitted to and approved by the Mount Royal University Human Research Ethics Review Board for research involving human subjects.

### Setting

Purposive sampling was used to recruit participants from a pool of 95 providers at 34 licensed center-based ECEC child care programs across Alberta, Canada that were a subset of a larger ongoing study investigating a health and wellness educational support program. Participants were employed at 14 different ECEC centers from six municipalities in Alberta, Canada. Geographically, center participation represented differing populations with most centers from large urban population locations (Table 1). Two centers were considered as rural or small population centers despite being governed under a larger municipality. Half of the ECEC centers identified as not-for-profit (50%) while the other half were for-profit.

**Table 1:** Participant and ECEC demographics

Variable	ECEC Centers	Early childhood educators
	(*n* = 14)	(*n* = 17)
Geographical location		
Large urban population center	9	
Medium population center	3	
Small population center	1	
Rural	1	
Center characteristics		
Not-for-profit	7	
For-profit	7	
Age, years, mean (SD)		42.65 (11.96)
Educator experience, years, mean (SD)		16.97 (13.92)
Female (%)		100
Education		
CDS^a^ Diploma		14
University degree		3

^a^Child development supervisor

### Participants

Participant criteria included: full-time employment at a center-based child care program operating with children in care during phase one or two of COVID restrictions, caring for preschool-aged children (2–5 years), present with children at lunchtime or snack time, and present during physical activity designated times throughout the week. Seventeen educators, all employed full-time consented to participate in the study. On average, the providers were aged 42.65 years and had 16.97 years of experience as a child care provider. More detailed educator demographics are presented in Table 1. Educators received a $25 gift card for participating and all participants provided informed consent before being interviewed.

### Data Collection

The semi-structured interview guide was developed using a recommended five-step process (Kallio et al., 2016). The present study met the criteria for using the semi-structured interviews as it provides participants the flexibility to fully share their perspectives on the complex experience of working as an educator during the pandemic (phase one). The interview questions were informed by previous nutrition and physical activity wellness literature and our work with the educators in a virtual community of practice project (phase two). The research team met to review and critique the preliminary interview guide followed by revision and reduction (phase three). The resulting guide was brought to nutrition, physical activity, and early learning educator experts for consultation and piloted with a community educator (phase four). Findings informed changes in protocol regarding clarifying wording for ease of understanding (phase five). Table 2 summarizes the interview questions.

**Table 2:** Semi-structured interview questions for nutrition and physical activity dimensions

Dimension	Example interview question
Physical activity	How has the current COVID pandemic impacted your physical activity routine with the children in your child care center? Do you feel that in the context of the COVID guidelines, you are able to keep the children active for recommended active daily minutes? What are the supports or barriers that you have experienced with regard to COVID that impact your ability to get children in your child care program active for the recommended active daily minutes?
Nutrition	How has the current COVID pandemic impacted your healthy eating routine with the children in your child care center? What are the supports or barriers that you have experienced with regard to COVID that impact teaching children about healthy eating at your child care center?
Open-ended	Is there anything else you would like to share?

### Interview Protocol

The research team conducted semi-structured, individual interviews with participants through an online communication platform (Google Meet) from July to August 2020, to comply with COVID-19 physical distancing requirements. Interviews were conducted by the research team community leads who were trained in one-on-one interviewing and held a peripheral established relationship with the participants. At the start of all interviews, the purpose and scope of the study, assured confidentiality, request to audio record the interview, and invitation to clarify or ask questions was explained to participants prior to conducting the interview. The interviews were transcribed verbatim by a transcriptionist and checked for accuracy by the authors.

### Data Analysis

All interview transcripts were imported into NVivo Mac (Release 1) to facilitate data analysis. Data was coded inductively through thematic analysis guided by a six-phase process as described by Braun and Clarke (2006) and Maguire and Delahunt (2017). First, the authors familiarized themselves with the data by thoroughly reading transcripts independently. Frequent meetings were held to discuss codes and the production of a codebook was developed collaboratively and iteratively. To develop consistency, each author coded the same selection of transcripts, allowing exploration and discussion of similarities and differences. Then, additional meetings were held to explore further concepts or missing codes. The collaborative process supported the identification of relationships between codes and trends in the data through discussion. In the final phase of data analysis, the authors met to explore and search the broader level themes and met several additional times to refine and name themes with coders while agreeing on definitions of each through verbal consensus.

### Rigor

The structured interview guide development, collaborative and recursive coding process, and quality of data contribute to the dependability, authenticity, credibility, and transferability of the results (Bradshaw et al., 2017; Braun & Clarke, 2006; Kallio et al., 2016; Kline, 2008). In the present study, attention to rigor and quality was addressed in three ways. First, a detailed description of the structured interview guide development process has been included in the data collection section. Second, a clear accounting of the collaborative and recursive coding has been included in the data analysis section. Third, a detailed identification of the participant characteristics and context has been included.

## Results

Results are presented in relation to the research questions as follows: impact of COVID-19 on the nutrition and physical activity environment in the early childhood education and care context; and impact of COVID-19 on educators’ teaching practices in support of children’s healthy eating and physical activity. Each of the main topics are discussed and their content is illustrated with quotes from participants.

### Nutrition and Physical Activity ECEC Environment—Impact of COVID-19

The educators’ perceptions of the impact of COVID-19 on the nutrition and physical activity environment within their ECEC center were expressed in two major themes.

#### Food Provision Transition

COVID-19 regulations changed food provision patterns in ECEC centers. Prior to March 2020, 13 (93%) of the 14 centers represented in the study provided food for children in their center, while only one center had family-provided food. After the reopening of ECEC centers during the COVID-19 pandemic, there was a large shift to families bearing the responsibility for food provision for their children during care time in ECEC centers (ten of fourteen centers). Table 3 summarizes the transition of food provision responsibility. In addition, educators commented on the food sent with the children, regarding the alignment or non-alignment with national food guide recommendations.

**Table 3:** The transition of food provision responsibility in response to COVID guidelines and assessment of alignment with food guide recommendations

Food provision	Context (frequency, % of total)	
	Pre-COVID	COVID
Food provided by ECEC centers	13 (93%)	4 (29%)
Families provide food	1 (7%)	10 (71%)
Educator’s assessment of food sent from home during COVID-19		
Food sent from home aligns with food guide recommendations (transcript examples include: vegetables such as carrots as well as fruit such as grapes & apples)		3 (30%)
Food sent from home does not align with food guide recommendations (transcript examples include: drink boxes, sugary muffins, chips, gummies, and sweets)		7 (70%)

In order to maintain physical distancing and capacity guidelines, educators indicated that the number of children that centers were able to accommodate was reduced. While some funding support was promised by government bodies to address this capacity reduction, overall, the financial impacts resulted in reduced staffing. Educators reported that when decisions were made on staffing priorities, often the loss of the center’s food preparation staff (i.e., chef) was one of the outcomes. This change was accompanied by the transition to family provided meals and snacks.

“We didn’t have a big enough budget to have a cook on site to make us healthy lunches and meals following the Canadian food guidelines, and so families were sending meals and they were, unfortunately, fairly terrible.” ~ Sophia.

Educators noticed that families were sending food that included items that both aligned and did not align with the healthy foods that the center had been serving. Previously the educators had some control and guidance over the frequency and availability of treats and sweets, and now these items were in the child’s lunch and this was occurring more frequently throughout the week.

“Well first our center used to have a chef who would make the food, and with the COVID we couldn’t do it, so now every kid is bringing their food, you know? And before it was pretty healthy, the things that we had in the center, and now parents are like, there is healthy food but there is also more sweets, and before we would not give them sweets at all. In the center we would never give them cookies, or chocolate, but no, now that they bring their lunches from home a lot of them have the gummies or the Bear Paws, you know, the cookies and they have more sweets than they had before, so it definitely has a big impact for us.” ~ Veronica.

Food handling guidelines were another reason that some ECEC centers transitioned to food provision by families. In order to manage hand washing guidelines and person-to-person transmission, food preparation and meal sharing was highly regulated. While centers were permitted to provide food service, some centers determined that the strict guidelines were too onerous and chose not to continue to provide food. These rules were communicated to families and educators.

“It was so hard because we weren’t allowed to provide lunches because that was one of the rules, so the parents were providing unhealthy snacks and we told them, we gave them resources, but it is like one of those things … then I don’t want to give them juice but that is all that they have, or Rice Krispies [square], or gummies, but that is what they have, you know?” ~ Anne.

The predominant message was that confections, sweets, cookies, and candies were included in the daily dietary intake of children as a result of transition to family-food provision.

#### Physical Activity Relief

The return to operation required ECEC centers to adopt new COVID-19 cleaning and physical distancing practices. Cleaning and disinfecting of shared toys and equipment was required as well as spacing out physical activity to avoid clustering. While educators were acutely aware of the new guidelines, children were eager to return to their usual interactions and patterns. Trying to keep children spaced apart was a challenging practice to implement. Educators found that taking children outside in larger spaces both aligned with physical spacing recommendations and eased the tension associated with trying to enforce physical distancing in their classroom spaces.

“We find that it is best to take the children out and that is the best way to keep them physically separated… we have been engaging our children very, like more often now physically and keeping them outdoor as much as we can.” ~ Parvati.

These conditions resulted in educators perceiving an increased physical activity level for the children compared to the pre-COVID environment.

“We actually have found that COVID has increased our physical activity exposure because we have had to get outside more. We are trying to keep the children spaced further apart, which means we are doing a lot more activities that involve physical motion, and we do luckily have a facility that has a lot of space in it.” ~ Nikita.

An expression of concern was frequently offered about how physical activity levels would be impacted by winter conditions when temperatures dropped and outside time became more challenging.

“Our physical activity is outside, and so we go outside much more than we ever have, and it is wonderful! I am thinking what happens if this is winter? This is not ideal. Wintertime is not going to be fun.” ~ Ophelia.

Generally, educators perceived that increased outdoor time and the resulting increased physical activity time for children, was a positive outcome both as a means to adhering to guidelines as well as providing relief for educators associated with COVID-19 regulation hypervigilance.

### Healthy Eating and Physical Activity Teaching Practices—Impact of COVID-19

The educators’ perceptions of the impact of COVID-19 on their teaching practices to support children’s healthy eating and physical activity were expressed in two major themes, with three nested subthemes.

#### Nutrition Education Disconnections

Pedagogy in early learning environments involves the incorporation of teaching strategies woven throughout daily routines. COVID-19 guidelines changed how food was handled in the center, which challenged educators in their efforts to create healthy eating experiences and the development of food literacy among children. The three subthemes related to pedagogical interruptions in nutrition education revolved around curriculum, life skills, and social connectedness.

#### Nutrition Education Disconnections: Curricular Interference

In an early learning curriculum framework approach, educators take their cues from children, and as children express curiosity or participate in food related activities, the healthy eating curriculum unfolds. The childcare center COVID-19 return to operation guidelines prohibited family-style meal services (Government of Alberta, 2020). Educators conveyed how this created interference for them in implementing and teaching a healthy eating curriculum.

“We were based off family-style dining together and they always fed off of each other. One would eat this and they would say, “Oh, you love it? Let me try it too!” and so we would eat the same thing and we would talk about it, and now we are all in a different space, you know?” ~ Isabelle.

Creating learning opportunities for children that connect food to their origins is an important component of nutrition education. Educators expressed that the COVID-19 return to operation guidelines prohibiting child participation in gardening and food preparation interfered with their teaching, learning, and program planning.

“We have a whole gardening section and things like that, so I sort of wish things would be lifted a bit so we could offer the fresh vegetables and fruit and things like that and have the children help us make salads and things like that, and cut it up and things, but obviously we can’t do any of that right now.” ~ Jayleen.

#### Nutrition Education Disconnections: Diminishing Life Skill Opportunities

Food skills that are practiced in the ECEC context support child development and teach children how to navigate a complex food system. This principle was important to many educators and they identified that the loss of these food related skill opportunities due to policy changes was a disconnect from their pedagogical philosophy.

“There is a concern in that part of our teaching of the children has always been life skills, so being able to serve themselves and knowing how much to each, and stuff like that, and that is just not possible right now, so that is challenging too.” ~ Sophia.

#### Nutrition Education Disconnections: Loss Of Social Connectedness

Educators expressed a sense of loss for sharing meals in a family-style setting with children in their care. Mealtime was identified as an important shared experience that offered the opportunity for co-constructed learning and conversations, and its loss due to physical distancing was difficult.

“Yeah, I would say it is a lot of emotional changes for the educators too because we can’t teach the way that we want to teach, and like, how I value children interacting with each other in kind of close quarters, but also sharing. I thought that mealtime was very important to us and we would sit and we would share the plate of food that the kitchen made for us or whatever, but now we can’t.” ~ Isabelle.

Educators recognized that adherence to COVID-19 regulations regarding food handling and serving behaviors created a disconnect with their ability to teach healthy eating curriculum and develop social connectedness within their classroom community.

#### Physical Activity Hurdles

Early childhood is a formative time for the development of movement skills and healthy, active behaviors. Educators play a pivotal role in supporting physical activity and teaching physical literacy. Adherence to COVID-19 guidelines disrupted usual ECEC centers daily activities which created hurdles for educators delivering movement experiences to support children’s healthy development. The subthemes under this category are related to space for physical activity, access to equipment, and loss of variety.

#### Physical Activity Hurdles: Limiting Physical Activity Space

Large indoor and outdoor environments are critical in providing adequate physical activity opportunities for preschoolers. Access to spaces that allow for gross motor activity is foundational to teaching and achieving movement. Educators indicated that increased cleaning requirements contributed to shrinking opportunities to access large spaces for physical activity.

“Yeah, so the only thing that has impacted us is in the sense that we are not using our gym area right now and so we can’t … just because there is a lot of cleaning, and we share the space with the church and it is just too much cleaning and set up, and take down, and all of that, so in that sense it has impacted it that way.” ~ Lydia.

#### Physical Activity Hurdles: Insufficient Equipment Access

Equipment designed to support physical activity, such as portable play equipment and floor markings, are effective approaches to supporting physical activity in young children. Educators commented that COVID-19 related cleaning and cohort guidelines led to limited equipment resources that they identified as a hurdle in supporting physical activity in their ECEC center.

“One of the things is that also now we have cohorts and the activities that they do, or the play equipment that they use has to be kept for one cohort, or it has to all be disinfected before another cohort can use it, so that has had an impact because it is having enough toys to add to physical activity. It is not feasible to clean everything ten minutes before the next group comes in. What we have had to do is each group has their own set of toys they can use outside for their own activities and they have their own play space, so that has been a challenge. Having enough outdoor equipment for them to utilize outside has been a challenge and so there is not a lot of support there to get that extra equipment that you would need.” ~ Nikita.

#### Physical Activity Hurdles: Loss of Variety

Centers avoided activities where children could not maintain the physical distancing COVID-19 guideline. Activities such as tag or playing catch were replaced with non-contact activities such as shadow tag (e.g., in which children touched other children’s shadows on the ground rather than having physical contact). Educators noted that their curriculum planning was impacted as a result of aligning with COVID-19 compliant permitted activities.

“Like sports week, we missed that last year, the children had great fun and they were going every day outside and preparing, according to their ages. The staff planned different activities, and this time we had limited things, limited choices, and everything is kind of limited now.” ~ Bhavana.

The COVID-19 guidelines also impacted the ability to bring experts into centers to provide enriched physical activity experiences such as sports ball, dance, or yoga.

“We usually have a yoga teacher who comes to the center to teach children about yoga, and now we have cancelled it and children miss that a lot and even the staff.” ~ Florence.

Educators noted that the loss of field trip exposure was a hurdle in providing new and varied activity exposure opportunities for children in their care.

“It has impacted where they can go outside to play and the field trip, because usually during the summer we are gone three days out of the week off somewhere, whether we are walking to different parks, or the zoo, Prince’s Island Park, or Rotary Park or Riley Park, or whatever, and we are restricted now. And also, we aren’t going to use transit to go anywhere just because the risk is too high. And even the Zoo—we are very close to the zoo and it is timed tickets and to get a time that would be convenient for our schedule and stuff, and then you are also limited as to what you can see and you are putting the children at risk being around a lot more people, and so it hasn’t impacted the actual physical play, but it has impacted the variety of the fun of doing other things.” ~ Praveena.

Physical activity is a large component of a preschooler’s life and in order to meet the guidelines considerable planning is required to integrate it throughout the day. Due to physical distancing and sanitizing requirements, educators found their ability to provide programming for physical activity compromised by shrinking spaces, dwindling equipment access, and variety of activities to engage the children in movement.

## Discussion

The purpose of the current study was to examine educators’ perspectives on how COVID-19 guidelines have impacted healthy eating and physical activity within the ECEC context. Educators identified experiencing changes across two major areas: (1) the environment, and (2) teaching practices. COVID-19 guidelines mediated nutrition changes and resulted in many centres transitioning from centre-provided food to family-provided food that precipitated an increase of ‘unhealthy food options’ available to children during their time in care. Conversely, a perceived increase in outdoor time, and thus increased physical activity, resulted from guideline adherence and was noted as a positive change that afforded educators with a measure of normalcy and relief. Educators expressed frustration with COVID-19 guidelines and their interference with teaching practices in nutrition education curriculum, life skills, and social connectedness. Teaching and promoting physical activity was found to be more difficult due to reduced space availability, play equipment options, and variety of activity experiences.

### COVID-19 and the Nutrition and Physical Activity Environment

The first key finding was the change to the eating environment as a result of COVID-19. Seventy percent of the ECEC centers in this study transitioned from preparing and serving meals to requiring families to supply food. ECEC centers that serve meals to children follow national food guide recommendations, serve all children the same meal, and have the ability to provide family-style meal service. Educators noted an increase in ‘unhealthy food options’ at mealtimes associated with this transition. In some cases, while healthy options were included, it was the addition of a ‘treat’ that accompanied the family-provided food that altered the assessment of food quality. These ‘treats’ were included in family-provided food more often than would have been provided in the ECEC food provision pattern. Educators also recognized that the change in quality of meals and snacks supplied by families could have occurred as a result of time constraints and financial challenges experienced by families. This aligns with recent research that indicates food insecurity has significantly increased as a result of COVID-19 (Adams et al., 2020).

The second key finding was the change in outdoor time in response to physical distancing recommendations, that is, children were exposed to supplementary outdoor time. Outdoor space is ideal for adhering to physical distancing and sanitization requirements. Educators indicated that outdoor time was a preferred activity due to minimal sanitizing and easy physical distancing adherence. The reduced cleaning required of outdoor time became a facilitator nudging educators outside more often as part of their daily routine with the perceived added benefit of providing some relief from COVID-19 related hypervigilance and enabling children to achieve or exceed the recommended daily physical activity targets. This aligns with research indicating a positive correlation between outdoor time and physical activity in early childhood (Coe, 2018; Herrington & Brussoni, 2015; Tremblay et al., 2015). This potential benefit is important as reports indicate that children in child care struggle to meet recommended daily physical activity targets (Kuzik, Clark, Ogden, Harber, & Carson, 2015; Tucker, 2008). Decreased outdoor playtime in response to pandemic related constraints was found with children and youth (5–17 years) that resulted in an overall reduction in physical activity time (Moore et al., 2020). Similarly, Caroll et al. (2020) found that physical activity in preschool-aged children decreased during COVID-19 due to a lack of adequate space and variety in available play equipment. In the current study, educators acknowledged warm summer weather as a key factor in the increased frequency and time spent outdoors. This was accompanied by concerns about providing children with similar outdoor time in the winter due to cold temperatures. Overall, an increased use of outdoor environments was a means to adhere to guidelines, which was identified to be valuable for both children and educators.

### COVID-19 and Educators’ Nutrition and Physical Activity Teaching Practices

Educators expressed an impact of COVID-19 guidelines on their nutrition and physical activity teaching practices. Educators experienced disruptions in their teaching of a nutrition curriculum, life skills, and social connectedness resulting in challenges to providing healthy eating experiences and food literacy development. Lost opportunities to incorporate healthy eating education in daily activities included the inability to introduce new foods, gardening, and food preparation activities. Children’s involvement in growing, preparing, and tasting healthy foods allows children to learn in a tangible way about the origins and life cycle of healthy food (Skelton et al., 2020). Additionally, the guideline prohibiting family-style meal service (FSMS) impacted educators’ capacity to teach children life skills associated with FSMS such as self-regulation and motor development. Allowing children to serve themselves is critical in learning hunger and satiation cues (Dev et al., 2014), and promoting gross and fine motor skill development (Benjamin Neelon & Briley, 2011; Fletcher et al., 2005). FSMS facilitates both feelings of social connectedness and learning opportunities, therefore, its removal hindered educator’s ability to provide children with quality mealtime interactions. FSMS has been shown to promote nutrition conversations with children, teaches mealtime manners, and models health promoting behaviours (Dev et al., 2014; Swinburn et al., 2013). Providing nutrition education and a healthy eating environment during early childhood contributes to children’s development of food literacy and influences lifelong healthy eating attitudes and behaviours (Parletta, 2014; Utter et al., 2018).

Educators’ teaching practices surrounding physical literacy have also been affected during the pandemic. Although time spent outside has increased, educators have still faced challenges to teaching and promoting physical activity within their ECEC center. The present study identified that stringent cleaning protocols and cohort separation limited access to both large indoor physical activity spaces and play equipment. Children’s indoor activity levels are positively associated with the size of available indoor space and access to play equipment that promotes active play (Bower et al., 2008; Henderson et al., 2015; Olesen et al., 2013). Shrinking indoor space and limited access to play equipment impacted educators’ ability to engage children in moderate-vigorous indoor physical activity, and this was identified as a concern for colder months when outdoor time would become less frequent. Prior to the COVID-19 pandemic, ECEC centers promoted physical literacy among preschoolers by offering a variety of activity exposures, this often-included field trips and community visitors facilitating activity (yoga, dance, ball). Educators reported that in order to minimize the risk of exposure to COVID-19, ECEC centers postponed all field trips and non-essential visitors. An outcome of these protective measures was the lack of activity variety educators rely on to engage children’s physical activity interest. Children’s movement during child care hours is positively associated with the frequency and quantity of provided physical activity opportunities (Gubbels et al., 2011). Dowda and colleagues (2004) identified that children who have the opportunity to go on field trips while attending child care tend to be more active than those without the same field trip opportunities. The COVID-19 guidelines limit preschooler’s exposure to various physical activities which may negatively impact children achieving movement guidelines in care. Overall, measures implemented in ECEC centers to protect children from COVID-19 have also impacted educators’ ability to promote physical activity.

Early childhood is a critical developmental period in the formation of healthy eating and active behaviors that track throughout the lifespan. Educators and a child’s environment have considerable influence on experiences that shape these behaviors. Findings from the present study reveal that COVID-19 guidelines directly impact ECEC environments and educators’ professional practice in their role to teach and promote healthy eating and active environments.

### Strengths and Limitations

This novel study explored educators’ perspectives of changes in ECEC eating and activity environments as a result of COVID-19 restrictions and the resulting impact on healthy child development. Second, the current study employed one-on-one interviews with a known interviewer, and this provided educators a safe space to share information in a pandemic context. Lastly, centers provided a wide representation of ECEC settings with differing characteristics across six cities encompassing large urban centres to rural communities. The similarity of expressed experiences across multiple municipalities demonstrates strength in the findings. A number of limitations should also be considered. First, the current study was conducted with educators from Alberta ECEC centers and might not reflect educators’ perceptions in different provinces or countries as different jurisdictions may have varied COVID-19 guidelines. Second, participants were recruited from a larger study on nutrition and physical literacy professional development training which may have resulted in participants having advanced understanding of these concepts compared to educators without similar training. Alternatively, this knowledge may have helped participants better identify their experiences that led to high quality interviews.

### Implications and Recommendations

Given our findings that COVID-19 guidelines had considerable impact on the teaching practices of educators, particularly in the healthy eating context, we suggest that public health services develop and distribute practical nutrition education teaching practices that demonstrate adaptations of best practices that align with pandemic guidelines. Our findings indicate that educators were taking children outside more than their usual pattern and felt that this increased child physical activity. Considering that children in care tend to not achieve physical activity recommendations, we suggest that a focus on outdoor time be promoted. Overall, the findings from this study can be used to help inform public health agencies that aim to assist educators in their ability to continue to provide care that will facilitate the healthy development of preschool children.

## Data Availability

Data and all other materials for this study are kept in the Department of Health and Physical Activity, Mount Royal University. The datasets generated during and/or analysed during the current study are not publicly available due to the terms of participant consent.
